# Feasibility, Diagnostic Accuracy, and Satisfaction of an Acute Pediatric Video Interconsultation Model in Rural Primary Care in Catalonia: Prospective Observational Study

**DOI:** 10.2196/82133

**Published:** 2026-01-26

**Authors:** Marta Castillo-Rodenas, Núria Solanas Bacardit, Clotilde Farràs Company, Queralt Miró Catalina, Laia Solà Reguant, Aïna Fuster-Casanovas, Francesc López Seguí, Josep Vidal-Alaball

**Affiliations:** 1 Centre d'Atenció Primària Cardona, Gerència d'Atenció Primària i a la Comunitat de la Catalunya Central, Institut Català de la Salut (ICS), Cardona, Barcelona, Spain; 2 Intelligence for Primary Care Research Group (I4PC), Fundació Institut Universitari per a la Recerca a l'Atenció Primària de Salut Jordi Gol i Gurina, Manresa, Catalonia, Spain; 3 Unitat de Recerca i Innovació Gerència d'Atenció Primària i a la Comunitat de la Catalunya Central, Institut Català de la Salut (ICS), Carrer de Soler i March, 6, CAP Bages, Manresa, Catalonia, 08242, Spain, 34 936930040; 4 Health Promotion in Rural Areas Research Group, Fundació Institut Universitari per a la Recerca a l'Atenció Primària de Salut Jordi Gol i Gurina, Manresa, Catalonia, Spain; 5 Unitat d'Innovació, Direcció de Qualitat, Processos i Innovació, Hospital Universitari Vall d'Hebron, Barcelona, Catalonia, Spain; 6 Grup de recerca en Serveis Sanitaris, Vall d'Hebron Research Institute, Barcelona, Catalonia, Spain; 7 eHealth Lab Research Group School of Health Sciences and eHealth Centre, Universitat Oberta de Catalunya (UOC), Barcelona, Catalonia, Spain; 8 Chair in ICT and Health Centre for Health and Social Care Research, University of Vic - Central University of Catalonia (UVIC-UCC), Vic, Catalonia, Spain; 9 Centre de Recerca en Economia i Salut (CRES), Universitat Pompeu Fabra (UPF), Barcelona, Catalonia, Spain; 10 Department of Medicine, Faculty of Medicine, University of Vic - Central University of Catalonia (UVIC-UCC), Vic, Catalonia, Spain

**Keywords:** interconsultation, pediatrics, primary health care, remote consultation, rural health services, telemedicine, video consultation

## Abstract

**Background:**

In Catalonia, Spain, pediatric primary care is undergoing restructuring, including greater integration of information and communication technologies. The adoption of digital health solutions has increased notably since the COVID-19 pandemic. In areas with limited availability of health care professionals, digital tools are a key strategy for facilitating access to services and ensuring continuity of care.

**Objective:**

This study aimed to evaluate the feasibility, diagnostic agreement, and satisfaction of users and professionals of an acute pediatric video consultation model, referred to as video interconsultation, that includes a synchronous remote physical examination and takes place between health care professionals.

**Methods:**

This was a 20-month prospective within-patient diagnostic accuracy study including 200 children (aged 0‐14 y) with acute conditions in rural primary care in Catalonia. A secure, closed, real-time, web-based, clinician-assisted video consultation platform enabled remote pediatric assessment—visual examination, audio auscultation via a digital stethoscope, and caregiver-reported symptoms—with a pediatrician remotely guiding a nurse physically present with the child. The intervention was compared, in all cases, with a standard in-person pediatric assessment as the reference standard. Outcomes were feasibility, diagnostic accuracy, and user and professional satisfaction. The platform was developed based on telemedicine usability and clinical safety principles.

**Results:**

Of the 200 children enrolled, remote video consultations were successfully completed in 64.5% (129/200) of cases. Diagnostic agreement with in-person assessment was 78.2% (129/165). Overall mean diagnostic accuracy across all diagnoses was 0.99 (95% CI 0.98‐1.00), with a mean specificity of 0.99 (95% CI 0.98‐1.00) and a mean sensitivity of 0.90 (95% CI 0.84‐0.95), varying by condition, with lower performance for pathologies requiring detailed physical examination. Overall, 95% (190/200) of users and 74% (148/200) of professionals reported a positive experience.

**Conclusions:**

The proposed pediatric video consultation model was feasible, accurate, and well accepted for managing a substantial proportion of acute pediatric conditions in primary care. Its implementation could improve access to medical care in rural areas and help reduce health care disparities. Further research is needed to support scalability and implementation in routine clinical practice.

## Introduction

### Background

In Catalonia, children and adolescents represent nearly 20% of the total population. These life stages are critical for development and have specific health needs and challenges. Health interventions during childhood and adolescence have both short- and long-term effects on adult health. Therefore, prevention, health promotion, and equitable access to high-quality pediatric care are essential priorities for the health system [1]. Ensuring appropriate care requires access to a pediatric referral team composed of pediatricians and specialized nurses [2].

When primary care teams include professionals with formal pediatric training, clinical practice becomes more efficient and better aligned with children’s needs. Appropriate prescribing improves (particularly of antibiotics), vaccination coverage increases, and unnecessary diagnostic tests and specialist referrals decrease [3]. Similarly, pediatric-trained nurses play a key role in primary care by promoting child health within the community and schools, supporting the management of pediatric demand, and contributing to improved overall quality of care [4].

However, many regions, particularly rural and underserved areas, face a shortage of pediatric specialists, which challenges the continuity and quality of pediatric health care by limiting timely access to diagnosis and treatment. According to the Catalan Pediatrics Society, all primary care pediatric positions in Catalonia are currently filled; however, more than one-third are occupied by general practitioners who, although not pediatric specialists, provide pediatric care in primary care settings [5]. This proportion has increased in recent years, and the geographic distribution of pediatric providers remains uneven, making recruitment especially difficult in rural areas [6]. Similar patterns have been reported in other European countries and in the United States [7,8].

In this context, digital health tools have emerged as promising strategies to support primary care teams, improve access to pediatric expertise, and reduce disparities between urban and rural populations.

In Catalonia, the primary care system already incorporates information and communication technologies to enhance communication between patients and health care professionals, including *eConsulta*—an asynchronous teleconsultation platform—complementing telephone consultations [9]. The use of video consultations, however, remains limited despite a temporary increase during the COVID-19 pandemic. Although still rarely used in routine primary care, this experience has prompted renewed interest in exploring their potential applications in daily clinical practice [10].

Simple digital devices can now be integrated into video consultations to enable remote physical examinations using a digital camera, video otoscope, and digital stethoscope [11]. Initially designed for home use by caregivers, these devices allow pediatricians to receive real-time clinical information through a virtual connection. Similar telemedicine solutions are already implemented in several European countries and the United States, mainly in private health care settings [12,13].

When combined with these digital tools, video consultations can facilitate real-time collaboration between health care professionals. In this model, a nurse physically present with the patient performs the remote examination under the pediatrician’s guidance, allowing both history taking and a basic remote physical examination to be conducted synchronously. As this interaction occurs between health care professionals, it is referred to as a video interconsultation.

This approach is particularly suitable for acute pediatric cases—commonly referred to as same-day or urgent visits—that require pediatric assessment within 48 hours. Implementing this model could enhance access to pediatric care in remote areas, promote territorial equity, and reduce unnecessary emergency department referrals.

However, the limited evidence on the use of digital tools such as video interconsultation in pediatric primary care highlights the need to develop and evaluate new technology-integrated models of care, especially in rural areas [14].

### Objectives

This study aimed to evaluate the feasibility of a synchronous acute pediatric video interconsultation model that integrates a remote physical examination and is conducted between health care professionals, one of whom is physically present with the patient, in the rural primary care setting of Catalonia.

The study also sought to assess diagnostic adequacy compared with in-person visits and satisfaction among users and health care professionals, considering quality of care, patient safety, and key influencing factors such as reason for consultation, patient age, and visit duration.

This pediatric video interconsultation model is hypothesized to be a feasible, diagnostically adequate, and well-accepted approach in rural primary care settings.

## Methods

### Study Protocol

The study protocol has been published in a separate publication [15].

### Study Design

This was a prospective observational diagnostic accuracy study conducted in a real-world primary care setting, without modification of routine clinical practice. Each participant underwent both the index test (video interconsultation) and the reference standard (in-person visit) during the same clinical encounter, enabling a within-participant comparison of diagnostic performance.

Blinding of participants, clinicians, or outcome assessors was not feasible due to the design: the same pediatrician performed both evaluations sequentially in the same visit, and therefore, all parties were aware of the modality used. As the intervention was limited to a single synchronous video consultation per episode, the study design did not involve repeated use or longitudinal tracking of digital engagement.

Standard in-person pediatric assessment was selected as the reference comparator because it represents current clinical practice and the diagnostic gold standard in primary care.

The study follows the STROBE (Strengthening the Reporting of Observational Studies in Epidemiology) guidelines, incorporates relevant STARD (Standards for Reporting Diagnostic Accuracy Studies) principles, and adheres to CONSORT-EHEALTH recommendations, an extension of the CONSORT (Consolidated Standards of Reporting Trials) statement for reporting digital health interventions [16-18].

### Setting and Period

The study was conducted within the primary care network of the Central Catalonia Health Region (Institut Català de la Salut) at the Cardona Primary Care Center by the pediatric care team. This rural area provides services to approximately 5000 residents, including approximately 800 children aged 0 to 14 years, with a population density of 68 inhabitants per square kilometer.

Data collection took place from June 7, 2023, to January 22, 2025. Diagnostic confirmation occurred immediately after the video consultation through the in-person reassessment; therefore, no additional follow-up was required.

No technological or operational changes occurred during the study that could have affected the feasibility or diagnostic performance of video interconsultations.

### Participants

Eligibility criteria included children aged 0 to 14 years presenting with acute conditions requiring care within 48 hours. Acute illness was defined as a condition requiring medical attention within 48 hours. Parental informed consent was mandatory.

The exclusion criteria were routine checkups, chronic condition follow-ups, emergencies requiring immediate in-person care, cases that could be managed autonomously by nursing staff, and an absence of informed consent.

### Sample Size and Sampling

**Figure 1.**
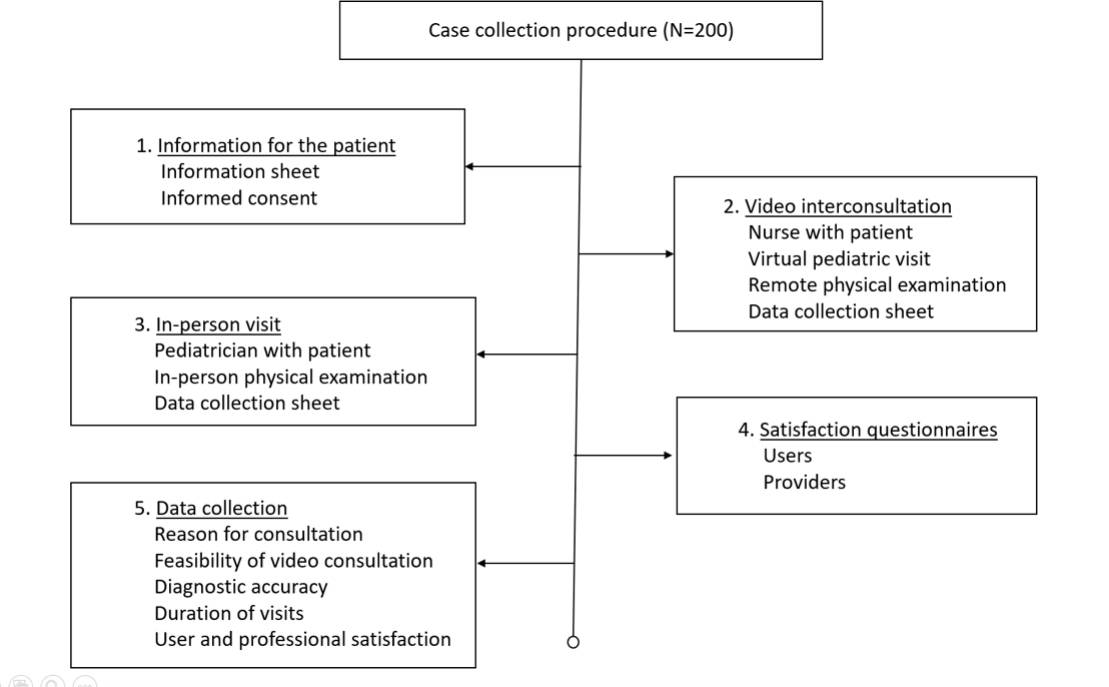
Sequential case collection process from recruitment and video interconsultation to in-person assessment, satisfaction evaluation, and data recording.

A convenience sample of all eligible cases was included. To ensure adequate power for the main objective, the minimum required sample size was estimated at 170 cases to assess the feasibility and diagnostic concordance of video interconsultations compared with in-person visits. The calculation was conducted using the GRANMO-DATARUS online tool, with a 95% CI, an 8% margin of error, and an anticipated 10% dropout rate [19]. A total of 200 cases were recruited. Participant flow is shown in Figure 1.

### Digital Health Intervention and Procedures

#### Information and Consent

Families were informed in person by the pediatric nurse during the visit, before enrollment.

#### Video Interconsultation (Index Test)

**Figure 2.**
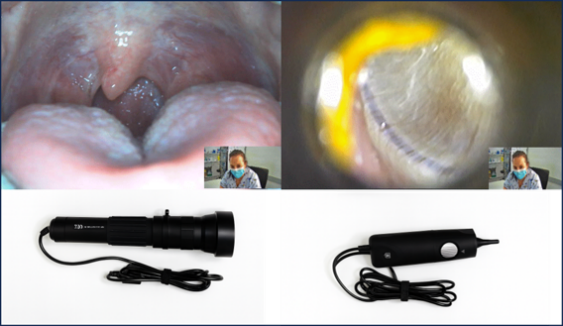
Images of the pharynx and tympanic membrane captured during the remote physical examination and the certified digital camera and digital video otoscope used in the study.

A secure, encrypted Microsoft Teams video call connected the pediatrician remotely with the onsite nurse and patient. With the pediatrician’s guidance, the nurse performed a physical examination using a digital camera, a video otoscope, and a digital stethoscope. A remote diagnostic impression was recorded. Example images are shown in Figure 2.

#### In-Person Reassessment (Reference Standard)

Immediately afterward, the same pediatrician performed a face-to-face evaluation and confirmed the final diagnosis.

#### Satisfaction Assessment

At the end of the visit, two brief questionnaires were administered: one for users (patients and families) and one for health care professionals (categorized as receiving pediatricians, assistant nurses, or observers).

### Data Collection

Data were collected using Microsoft 365 Forms via encrypted institutional accounts and stored on secure health services servers. All participants were exposed; no comparison group was included. As this trial was conducted in a rural primary care environment, all video consultations were mediated by trained health care staff.

A total of three data collection tools were used: (1) a clinical form, (2) a professional satisfaction questionnaire (validated Catalan version of the Health Optimum Telemedicine Acceptance Questionnaire) [20], and (3) a user satisfaction questionnaire (adapted from the Northern Saskatchewan Telehealth Network) [21].

No privacy breaches or adverse events occurred. Technical issues were managed by redirecting participants to an in-person assessment when needed.

### Variables

The following variables were collected to describe the characteristics of the sample and to assess the feasibility, diagnostic adequacy, and satisfaction associated with pediatric video consultations:

- Sociodemographic variables

  - Age: grouped into five 3-year intervals
  - Sex: female, male, or nonbinary

- Clinical variables

  - Reason for consultation: recorded individually and categorized by affected system or area (respiratory; otorhinolaryngology or ear, nose, and throat; gastrointestinal; infectious diseases; dermatology; musculoskeletal; ocular; and other)
  - Diagnosis: recorded individually and categorized using the same classification

- Feasibility variables

  - Feasibility: feasible or infeasible based on the ability to establish a safe and appropriate diagnosis. Patient safety indicators are included in feasibility, defined as the ability to reach a correct diagnosis without causing harm.
  - Duration of both consultations (in minutes)
  - Limiting factors of nonfeasibility: classified into 6 groups (need for in-person physical examination, telematic auscultation difficulty, camera visualization difficulty, video otoscope visualization difficulty, urgent demand, and lack of patient cooperation)

- Diagnostic adequacy variables

  - Diagnostic concordance: correct or incorrect, using the in-person visit as the gold standard
  - Sensitivity, specificity, and accuracy of the video interconsultation

- Satisfaction variables

  - Professional satisfaction score
  - User satisfaction score

### Outcomes Measures

The primary outcomes were the feasibility and diagnostic accuracy of the pediatric video interconsultation model compared with in-person visits, as well as satisfaction levels among health care professionals and users.

The secondary outcomes included factors limiting feasibility (reason for consultation, patient age, and visit duration) and potential barriers identified during the implementation process.

### Statistical Analysis

Categorical variables were summarized using absolute frequencies and percentages, whereas continuous variables were described using means and SDs. Associations between categorical variables were analyzed using the Pearson chi-square test or Fisher exact test when expected cell counts were less than 5. For continuous variables, comparisons were made using the *t* test or, when normality assumptions were not met, the Mann-Whitney *U* test.

Diagnostic performance of video consultations was evaluated using sensitivity, specificity, and overall diagnostic accuracy, considering the in-person assessment as the reference standard. CIs for accuracy estimates were calculated using the Wilson method. Diagnostic concordance was assessed using the Cohen κ coefficient, and Gwet’s first-order agreement coefficient (AC1) was additionally computed to account for potential prevalence and bias effects. The binomial test was used to analyze the type and direction of diagnostic disagreements. When cell counts were small, estimates were interpreted with caution due to limited statistical power.

All estimates were reported with 95% CIs, and statistical significance was set at *P*<.05. Statistical analyses were performed using R software (version 4.0.3; R Foundation for Statistical Computing).

### Ethical Considerations

This study was approved by the Ethical Committee for Research in Medicines of the Jordi Gol i Gurina Primary Care Research Institute (Barcelona, Spain; registration number 22/236-P; March 8, 2023).

As participants were minors, written informed consent from their parents or legal guardians was mandatory. Families received both oral and written information about the study at the time of the visit, before providing consent. The information included the study purpose, procedures (first, a video consultation performed with the pediatric nurse onsite while the pediatrician participated remotely, followed by a conventional in-person visit with the same pediatrician), eligibility criteria, potential risks, confidentiality, and lawful data protection. Participation was voluntary, and withdrawal was possible at any time without consequences for clinical care. Contact details of the principal investigator were provided to address any present or future questions. No images or videos were recorded during the video consultations, except in isolated cases where photographs of clinical findings were taken with explicit informed consent and used exclusively for research or training purposes, ensuring that patients could not be identified. Participants did not receive any financial or other form of compensation for participation in the study.

The telemedicine assessment was supplementary to standard in-person clinical care, ensuring that no diagnostic or therapeutic decisions relied solely on the digital evaluation. Video calls were conducted using secure and encrypted systems to ensure the confidentiality of clinical information.

All researchers signed a confidentiality agreement concerning the treatment and use of study data. No direct personal identifiers were collected, and all data were pseudonymized and processed confidentially. Access to the data was restricted exclusively to the research team. The project database is hosted on secure servers of the Primary Care Management and Community of the Catalan Health Institute (Institut Català de la Salut), which acts as the data processor. Data retention is planned for 10 years, and no international data transfers are anticipated.

The research team will only use the coded database for scientific purposes (eg, journal articles, scientific reports, and book chapters). The study was conducted in full compliance with the ethical principles of the Declaration of Helsinki (1964) and its latest amendment (Fortaleza, 2013), as well as with the European General Data Protection Regulation (GDPR EU 2016/679) and Spanish Organic Law 3/2018 on the Protection of Personal Data and Guarantee of Digital Rights.

## Results

### Participant Characteristics

A total of 200 pediatric video interconsultations for acute conditions were conducted in rural primary care. All diagnosis-related data were complete for all included cases; no missing diagnostic data were present. The sample distribution by age and sex, along with the main reasons for consultation, is summarized in Table 1. These reasons are provided both individually and grouped by system to facilitate analysis: respiratory, otorhinolaryngology or ENT, gastrointestinal, infectious diseases, dermatology, trauma, ocular, and other. The most prevalent reasons for consultation were respiratory, otorhinolaryngologic, and dermatologic conditions, with cough, earache, and skin lesions being the most frequent symptoms, followed in frequency by fever and odynophagia. Specific reasons for consultation for each organ system are provided in Multimedia Appendix 1.

**Table 1.** Characteristics of the sample (N=200).

Characteristics	Values
Patient sex, n (%)	
Female	98 (49)
Male	102 (51)
Patient age (y), n (%)	
0‐2	38 (19)
3‐5	44 (22)
6‐8	48 (24)
9‐11	37 (18.5)
12‐14	33 (16.5)
Grouped consultation reasons, n (%)	
ENT^a^	57 (28.5)
Respiratory	55 (27.5)
Dermatology	23 (11.5)
Infectious	21 (10.5)
Trauma	13 (6.5)
Gastrointestinal	11 (5.5)
Ocular	7 (3.5)
Other	13 (6.5)
Duration (min^b^), mean (SD)	
Video interconsultation	7.13 (3.85)
In-person visit	3.96 (1.57)
Feasibility video interconsultation, n (%)	
Feasible	129 (64.5)
Infeasible	71 (35.5)

a ENT: ear, nose, and throat.

b *P* value <.001 based on an independent samples Student *t* test.

Regarding consultation duration, video interconsultations had a significantly longer mean duration (*P*<.001) of 7.13 (SD 3.85) minutes compared with in-person visits, which had a mean duration of 3.96 (SD 1.57) minutes.

### Feasibility

Video interconsultation was feasible in 129 (64.5%) of the 200 cases. In these visits, the video interconsultation could be completed appropriately, providing the necessary data to issue a reliable diagnosis while maintaining quality of care and patient safety.

In 71 (35.5%) cases, video interconsultation was not feasible. The causes were analyzed, and the most frequent cause of infeasibility was the inability to perform a complete physical examination electronically, requiring redirection to an in-person visit (27/71, 38%). Other limitations included difficulties in interpreting online auscultation (18/71, 25.4%), problems viewing images obtained with the digital camera (12/71, 16.9%), and problems with the video otoscope (10/71, 14.1%). In 5 (7%) cases, the consultation was urgent and could not be completed via video, and in 2 (2.8%) cases, the patient’s lack of cooperation prevented completion. In 3 (4.2%) cases, there were combined technical difficulties, with simultaneous problems in interpreting images from both the digital camera and the video otoscope or in the quality of auscultation through the electronic stethoscope (Table 2).

**Table 2.** Reasons for infeasibility of video interconsultations and corresponding frequencies (N=71).

Reasons for video interconsultation infeasibility^a^	Frequency, n (%)
Need for an in-person physical examination	27 (38)
Difficulty with remote auscultation	18 (25)
Limited visibility through digital camera	12 (17)
Limited visibility through video otoscope	10 (14)
Urgent consultation required	5 (7)
Lack of patient cooperation	2 (3)

a Multiple reasons were reported in 3 cases.

A bivariate analysis was conducted to identify demographic and clinical variables potentially associated with the feasibility of video interconsultations.

Categorical variables, including patient sex, age group, diagnostic adequacy, and the clinical category of the consultation reason, were compared between feasible and nonfeasible cases using the chi-square test. A *P* value <.05 was considered statistically significant. No significant associations were found between feasibility and the patient’s sex or age group.

However, a statistically significant relationship was observed between feasibility and the clinical category of the consultation reason. Consultations related to gastrointestinal, musculoskeletal, and other conditions showed a higher proportion of nonfeasible cases, whereas those for dermatologic, ocular, and otorhinolaryngologic conditions demonstrated a higher proportion of feasible cases.

Diagnostic adequacy was also significantly associated with feasibility, as all cases classified as feasible presented correct diagnostic agreement (*P*<.001; Table 3).

**Table 3.** Bivariate analysis of main variables by feasibility of telemedicine visits.

Variables	Infeasible (n=71)	Feasible (n=129)	*P* value^a^
Patient sex, n (%)			.21
Male	30 (42.3)	68 (52.7)	
Female	41 (57.7)	61 (47.3)	
Patient age (y), n (%)			.19
0‐2	15 (21.1)	23 (17.8)	
3‐5	9 (12.7)	35 (27.1)	
6‐8	20 (28.2)	28 (21.7)	
9‐11	13 (18.3)	24 (18.6)	
12‐14	14 (19.7)	19 (14.7)	
Diagnostic agreement, n (%)			<.001
Correct	35 (49.3)	129 (100)	
Incorrect	36 (50.7)	0 (0)	
Reason for consultation, n (%)			<.001
Respiratory	20 (28.2)	35 (27.1)	
ENT^b^	12 (16.9)	45 (34.9)	
Gastrointestinal	10 (14.1)	1 (0.8)	
Trauma	9 (12.7)	4 (3.1)	
Other	9 (12.7)	4 (3.1)	
Infectious	8 (11.3)	13 (10.1)	
Dermatology	3 (4.2)	20 (15.5)	
Ocular	0 (0)	7 (5.4)	

a *P* values calculated using the *χ* ² test.

b ENT: ear, nose, and throat.

### Accuracy

Regarding diagnostic accuracy, diagnoses were grouped by system, using the same eight categories as the reasons for consultation: respiratory, otorhinolaryngology, gastrointestinal, infectious diseases, dermatology, trauma, ocular, and other. In all 129 cases in which video interconsultation was feasible, diagnostic concordance with the in-person visit was observed, as this was considered inherent to the concept of feasibility.

**Figure 3.**
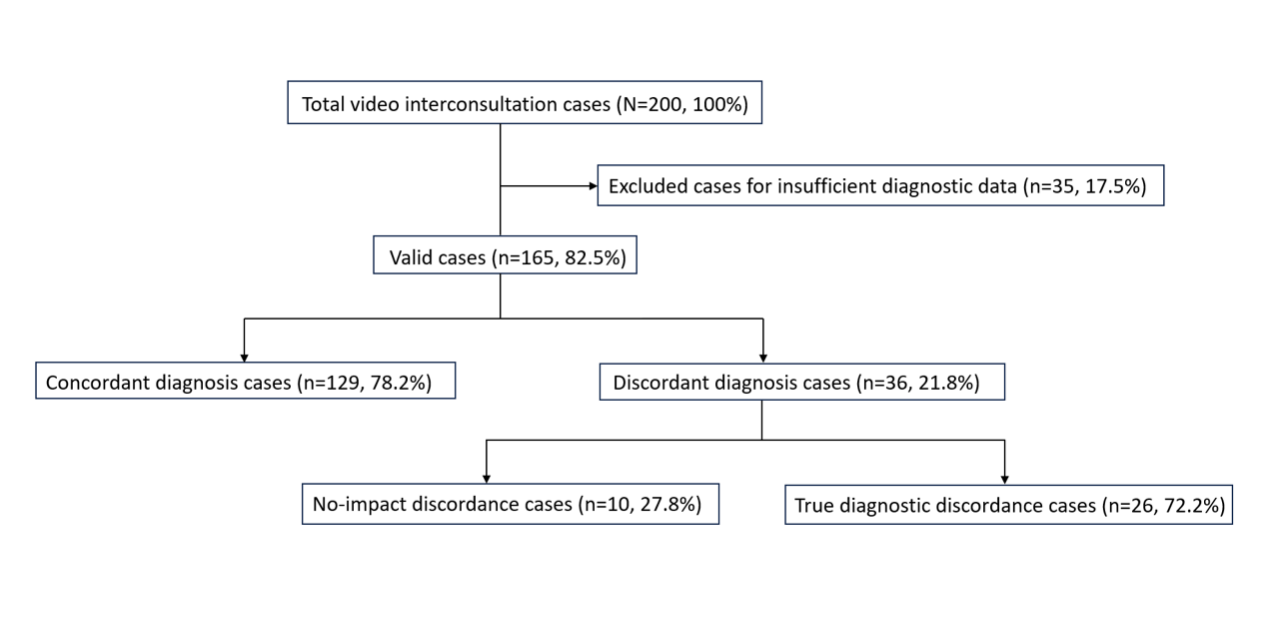
Flow of participants throughout the study according to diagnostic agreement analysis. The different percentages for all subgroups are indicated in relation to the corresponding group.

In contrast, among the 71 nonfeasible cases, diagnostic discrepancies between the video interconsultation and the subsequent in-person visit were identified in 36 instances. To calculate diagnostic agreement, 165 valid cases were included, as 35 cases were excluded because of insufficient data for diagnostic evaluation. Diagnostic concordance between the video interconsultation and the in-person assessment was observed in 129 (78.2%) of 165 feasible cases. In the remaining 36 (21.8%) cases, the diagnoses differed between the 2 assessment modalities. Figure 3 shows the flow of participants through the study and the diagnostic concordance analysis.

Cohen κ coefficient for telematic–in-person diagnostic concordance was 0.36, indicating fair agreement. However, given the unbalanced distribution of diagnostic categories, Gwet’s AC1 coefficient was also computed, yielding a value of 0.67, which indicates substantial agreement, according to the Landis and Koch scale.

Among the 36 (21.8%; total 165) cases of showing a diagnostic discrepancy between the telematic and in-person assessments, 10 (27.8%) cases were attributed to the need for a complete physical examination to establish an accurate diagnosis. The distribution of these cases was as follows: 5 involved the musculoskeletal system, 3 were related to the gastrointestinal system, 1 was related to infectious diseases, and 1 was related to other conditions.

Of the remaining 26 (72.2%) discordant cases, the type of diagnostic discrepancy and its distribution by organ system and specific diagnosis were analyzed (Table 4). Most discrepancies were underdiagnoses (23/26, 88.5%), while 5 (19.2%) cases represented overdiagnoses. The exact binomial test showed that the proportion of underdiagnoses was significantly greater than 50% (95% CI 69.8%‐97.6; *P*<.001).

**Table 4.** Types and frequencies of diagnostic discrepancies between video interconsultations and in-person visits (n=26).

Discrepancy type (video interconsultation vs in person)	Cases^a^ n (%)
Underdiagnosis of respiratory conditions	
Bronchospasm	6 (23.1)
Respiratory superinfection	4 (15.4)
Bronchiolitis	1 (3.8)
Overdiagnosis of respiratory conditions	
Bronchospasm	2 (7.7)
Underdiagnosis of ENT^b^ conditions	
Acute otitis media	3 (11.5)
Herpangina	3 (11.5)
Otitis externa	2 (7.7)
Streptococcal pharyngitis	1 (3.8)
Dental abscess	1 (3.8)
Overdiagnosis of ENT conditions	
Acute otitis media	3 (11.5)
Underdiagnosis of dermatology conditions	
Atopic dermatitis	1 (3.8)
Scarlet fever	1 (3.8)

a One case involved both underdiagnosis in the respiratory system and overdiagnosis in ENT. Another case showed both underdiagnosis and overdiagnosis within ENT.

b ENT: ear, nose, and throat.

Of the 23 (88.5%; total 26) underdiagnosed cases in the video interconsultation assessment, 11 (%) corresponded to the respiratory system, 10 (%) corresponded to otorhinolaryngology, and 2 (%) corresponded to dermatology. The distribution of underdiagnoses by organ system did not differ significantly from a uniform distribution (Fisher exact test, *P*=.08).

Regarding overdiagnosis, of the 5 (19.2%) detected cases, 2 (%) involved the respiratory system, and 3 (%) involved the otorhinolaryngology system. Given the small sample size, no specific pattern could be confirmed or ruled out, and the Fisher exact test also showed no significant deviation from a uniform distribution (*P*≈.6).

In 2 cases, both underdiagnosis and overdiagnosis occurred simultaneously and were therefore classified in both categories. In 1 case, overdiagnosis involved an otorhinolaryngologic condition and underdiagnosis a respiratory one; in the other, both diagnoses were within otorhinolaryngology, where acute otitis media was incorrectly diagnosed instead of otitis externa during the video interconsultation.

The accuracy, sensitivity, and specificity of each diagnosis obtained via video interconsultation were estimated using the in-person diagnosis as the gold standard. Importantly, the mean diagnostic accuracy of video interconsultations across all conditions was 0.99 (95% CI 0.98‐1.00). The mean overall specificity was 0.99 (95% CI 0.98‐1.00), and the mean overall sensitivity was 0.90 (95% CI 0.84‐0.95).

The diagnostic performance metrics for each clinical category, organized by organ system, are summarized in Table 5. A high level of diagnostic agreement was observed between video and in-person consultations across all categories, with accuracy values exceeding 0.92 and ranging from 0.93 to 1.00. Video interconsultations demonstrated the best performance for otorhinolaryngologic, dermatologic, and trauma-related conditions, which also showed high sensitivity and specificity. In contrast, respiratory diagnoses had lower sensitivity (0.68, 95% CI 0.51‐0.82), suggesting a potential risk of diagnostic underestimation during video interconsultations. Detailed diagnostic performance metrics by specific condition, along with the corresponding frequency distribution, are provided in Multimedia Appendix 2. CIs should be interpreted with caution in categories with small sample sizes.

**Table 5.** Diagnostic frequency by system in video and in-person consultations, with corresponding accuracy, sensitivity, and specificity (N=200).

Organ system	Video visit, n (%)	In-person visit, n (%)	Accuracy (95% CI)	Sensitivity (95% CI)	Specificity (95% CI)
ENT^a^	106 (53)	96 (48)	0.92 (0.87‐0.95)	0.97 (0.91‐0.99)	0.88 (0.80‐0.93)
Respiratory	28 (14)	38 (19)	0.93 (0.89‐0.96)	0.68 (0.51‐0.82)	0.99 (0.96‐1.00)
Dermatology	21 (10.5)	22 (11)	0.99 (0.97‐1.00)	0.95 (0.77‐1.00)	1.00 (0.98‐1.00)
Trauma	12 (6)	13 (6.5)	0.99 (0.97‐1.00)	0.92 (0.64‐1.00)	1.00 (0.98‐1.00)
Gastrointestinal	10 (5)	9 (4.5)	0.99 (0.97‐1.00)	1.00 (0.66‐1.00)	0.99 (0.97‐1.00)
Other	10 (5)	9 (4.5)	0.98 (0.96‐1.00)	0.89 (0.52‐1.00)	0.99 (0.96‐1.00)
Infectious	7 (3.5)	7 (3.5)	0.99 (0.96‐1.00)	0.86 (0.42‐1.00)	0.99 (0.97‐1.00)
Ocular	7 (3.5)	7 (3.5)	1.00 (0.98‐1.00)	1.00 (0.59‐1.00)	1.00 (0.98‐1.00)

a ENT: ear, nose, and throat.

### Professional Satisfaction

Professional satisfaction, assessed through the validated Catalan version of the Health Optimum Telemedicine Acceptance Questionnaire, was analyzed by comparing responses across three professional groups—receivers (pediatricians), assistants (nurses), and observers (residents and students)—using the chi-square tests (Table 6).

In terms of perceived quality, 74% (148/200) of professionals rated the video interconsultation as equal to or higher than in-person care, whereas 26% (52/200) perceived it as lower. This assessment differed significantly among professional groups (*P*<.001). Nursing assistants expressed the most favorable opinions (42/47, 89.4%, positive ratings), similar to observers (41/46, 89.1%), whereas receivers (pediatricians) were less favorable, with 50.5% (54/107) positive and 43% (46/107) negative ratings.

Overall, 94.5% (189/200) of participants believed that telemedicine could have a positive impact on patient health, 4.5% (9/200) thought it had no influence, and 1% (2/200) considered that it might worsen patient outcomes. When analyzed by professional group, receivers reported in 99.1% (106/107) of visits that telemedicine could improve patient health, compared with 78.7% (37/47) among assistants and 100% (200/200) among observers. Among assistants, 4.3% (2/47) indicated that telemedicine could negatively affect patients’ health.

With respect to the continuity of telemedicine use, 83.4% (166/199) of professionals indicated that improvements were needed in infrastructure or organization. Receivers were particularly likely to request such improvements (96/107, 89.7%), compared with assistants (15/47, 31.9%) and observers (7/45, 15.6%; one observer did not provide a response to this item).

Differences among professional groups were statistically significant for both perceived quality (*P*<.001) and willingness to continue using telemedicine (*P*=.004).

**Table 6.** Health care professionals’ satisfaction with telemedicine and video interconsultation.

Opinion professionals	Total (N=200), n (%)	Assistant (nurse; n=47), n (%)	Receiver (pediatrician; n=107), n (%)	Observer^c^ (other professionals^a^; n=46), n (%)	*P* value^b^
Perceived quality					<.001
Very good	63 (31.5)	25 (53.2)	12 (11.2)	26 (56.5)	
Good	74 (37)	17 (36.2)	42 (39.3)	15 (32.6)	
Fair	11 (5.5)	1 (2.1)	7 (6.5)	3 (6.5)	
Poor	35 (17.5)	3 (6.4)	31 (29)	1 (2.2)	
Very poor	17 (8.5)	1 (2.1)	15 (14)	1 (2.2)	
Perceived impact					<.001
No	9 (4.5)	8 (17)	1 (0.9)	0 (0)	
Yes, positive	189 (94.5)	37 (78.7)	106 (99.1)	46 (100)	
Yes, negative	2 (1)	2 (4.3)	0 (0)	0 (0)	
Intent to continue^c^					.004
Unchanged	33 (16.6)	15 (31.9)	11 (10.3)	7 (15.6)	
Improved	166 (83.4)	32 (68.1)	96 (89.7)	38 (84.4)	

a Other professionals included residents and students.

b *P* values were calculated using the *χ*² test.

c One observer did not provide a response to this item.

### User Satisfaction

User experience, measured using a survey adapted from the Telehealth Network Questionnaire (Northern Saskatchewan Telehealth Network) and completed by the accompanying adults responsible for the child, was rated as very good in 74.5% (149/200) of the 200 cases and good in 20.5% (41/200). A total of 4.5% (9/200) of participants described the experience as poor, and 0.5% (1/200) of participants described the experience as very poor (Table 7).

In addition, 92% (184/200) of families reported that they would be willing to repeat the video interconsultation in the future (142/200, 71%, very likely, and 42/200, 21%, likely), whereas only 8% (16/200) expressed reluctance (12/200, 6%, unlikely and 4/200, 2%, very unlikely).

To explore whether these perceptions were shared by health care professionals, a correlation analysis between user and professional satisfaction for each visit revealed a weak but statistically significant positive correlation (*r*=0.182, 95% CI 0.044‐0.312; *P*=.009).

**Table 7.** Users’ satisfaction with telemedicine and video interconsultation (N=200).

Opinion users	Total, n (%)
Overall quality	
Very good	149 (74.5)
Good	41 (20.5)
Poor	9 (4.5)
Very poor	1 (0.5)
Willingness to repeat	
Very likely	142 (71)
Likely	42 (21)
Unlikely	12 (6)
Very unlikely	4 (2)

## Discussion

### Principal Findings

This study evaluated whether a synchronous video consultation model—including remote physical examination guided by a pediatrician and supported by an onsite pediatric nurse—is a feasible, clinically appropriate, and well-accepted approach to managing acute pediatric conditions in rural primary care in Catalonia.

Feasibility was achieved in nearly two-thirds of cases, allowing completion of a safe and adequate remote assessment. Among feasible cases, diagnostic performance was high, with substantial agreement between telemedicine and in-person evaluations. Video consultations also demonstrated high diagnostic accuracy and sensitivity, along with near-optimal specificity. However, performance varied by clinical condition, suggesting that limitations inherent to remote assessment (eg, reduced ability to detect subtle clinical signs) may reduce sensitivity in certain scenarios.

Process outcomes further supported implementation: satisfaction was very high among families and positive among health care professionals, and consultation duration remained acceptable despite the inclusion of a remote physical examination.

Together, these findings indicate that—when appropriate feasibility and safety criteria are met—this telemedicine model can effectively complement, rather than replace, in-person pediatric care in underserved regions, thereby contributing to improved health equity and accessibility.

Furthermore, these findings could be applicable to other pediatric primary care settings with similar digital infrastructure and staffing resources. However, as this was an observational, single-center study without randomization, these findings should be interpreted with caution regarding their external validity.

### Comparison With Prior Work

These results are consistent with the current literature suggesting that telemedicine in pediatrics can achieve high diagnostic validity when applied under well-defined conditions. That said, most previous studies were not conducted in primary care settings, did not address acute conditions, and primarily examined video consultations between professionals and patients, rather than between professionals themselves [22-24]. There is limited evidence on video consultations that incorporate physical examinations. In pediatrics, Wagner et al [25] found that remote physical examination using medical devices similar to those used in this study was comparable to in-person assessment. Their findings align with ours, showing high diagnostic accuracy for otoscopy, oropharyngeal evaluation, and dermatological examination, but lower accuracy for assessing abdominal pathology.

Several studies have found that the effectiveness of video consultations is particularly high for diagnoses that rely primarily on medical history and visual assessment, such as upper respiratory tract infections (eg, pharyngitis and otitis) or dermatologic lesions [26,27]. In contrast, conditions that require a full physical examination, such as abdominal pain, which necessitates palpation, or headache, which may call for a neurological evaluation, may show reduced sensitivity, as also observed in this study [25,28].

This limitation may increase the risk of incomplete or inaccurate diagnoses, as reflected in the 21.8% (36/165) of cases that showed diagnostic discordance between virtual and in-person visits. While most discrepancies were classified as underdiagnoses or overdiagnoses, some involved mixed errors. Several cases required referral to in-person care to complete the physical examination and establish a reliable diagnosis, underscoring the complexity of remote clinical assessment. Furthermore, in some cases classified as infeasible, the online and in-person diagnoses were consistent, and the infeasibility was attributed to other limitations. In pediatric care, particularly for younger children, a comprehensive physical examination is often performed regardless of the presenting complaint. These findings underscore the need for triage and feasibility criteria tailored to the specific characteristics of each patient and condition to optimize the safety and effectiveness of video interconsultations. They also highlight the importance of establishing clear clinical guidelines to ensure the safe, high-quality use of video interconsultations in primary care pediatrics.

Pediatric patients with specialty conditions, such as rheumatologic, cardiologic, or endocrinologic disorders, were not included, as no such cases were seen during the study period. However, a few neurology and gynecology cases were included in the “other” category due to their small number. It is worth noting that telemedicine has also proven useful for the follow-up of pediatric rheumatic diseases, particularly in rural areas and during the COVID-19 pandemic [29].

Regarding consultation duration, a 2022 review by the Catalan Agency for Health Quality and Evaluation reported that video or telephone consultations are typically 1.5 to 4 minutes shorter than in-person visits [30]. In contrast, in this study, video interconsultations lasted nearly twice as long as in-person visits, likely due to the inclusion of a physical examination, which extended the consultation time. Additionally, this model involved 2 health care professionals. While it may enhance accessibility, it also requires greater resource allocation in terms of time and staffing.

Regarding users’ perceptions of telemedicine, the findings of this study indicate a high level of satisfaction, consistent with previous research [31]. The main reasons families expressed concerns about remote interconsultations were technical issues related to sound, connectivity, and image quality, as well as fears that in-person care might be replaced by virtual services. Other reasons, such as a perceived lack of safety, were infrequent. In any case, it is essential to provide families with clear and transparent information so they can make informed decisions about using this technology. Ultimately, they remain at the center of care [32].

With regard to health care professionals’ perspectives on telemedicine, the results indicate that professionals acknowledge the value of pediatric video interconsultations, and most consider them beneficial for patient health. However, differences among professional groups suggest that perceptions vary depending on each participant’s clinical role and expectations.

These findings are consistent with those of Martín-Masot et al [33], who analyzed the views of Spanish pediatricians following the rapid digitalization of health care delivery during the COVID-19 pandemic. In their study, most pediatricians regarded digital consultations as time-efficient and a valuable resource, aligning with the present results. Among nurses, the majority reported that the quality of video consultations was equal to or better than that of in-person visits. These results align with the findings of Navarro-Martínez et al [34], who reported that telenursing is positively perceived in routine clinical practice. However, most health care professionals in this study identified the need to improve the application of telemedicine in clinical settings, particularly in terms of technology, organizational processes, and bioethical considerations. This observation is echoed in a study conducted in Catalonia by Vidal-Alaball et al [35]. Similarly, other studies, such as that by Inoue et al [36] in Japan, have reached the same conclusions. In this context, it is essential to train professionals not only in the use of digital tools, but also in what Finkelstein et al [37] refer to as “webside manner,” a more effective approach to online clinical communication.

Furthermore, although telemedicine can improve communication between doctors and patients and help reduce health care costs, it may compromise the quality of care, therapeutic effectiveness, and patient safety if not implemented properly [38]. Therefore, legislation and bioethical frameworks must evolve to accommodate these emerging models of care [39].

### Limitations

This study has several limitations. First, there is a potential risk of diagnostic inaccuracy in video consultations compared with in-person visits, especially for conditions that require a direct physical examination, such as abdominal pain, trauma, or headache. Technical issues affecting image or audio quality may also hinder adequate remote assessment. Additionally, some diagnostic subgroups were small, which reduced the precision of accuracy estimates and widened the CIs.

Confirmation bias may have occurred because the same pediatrician conducted both the telemedicine and in-person evaluations. A role bias may also have occurred, as those receiving telemedicine may have different experiences from those delivering or observing it. If recipients represent the majority, as in this case, overall satisfaction may primarily reflect their perspective. Likewise, observers may have overemphasized methodological aspects while underestimating the actual user experience.

Another limitation is a possible social desirability bias, as the project was conducted by the patients’ regular and trusted pediatrician and nurse. This relationship of trust may have influenced participants’ responses, although no intentional intervention or influence was exerted during data collection.

Operational limitations should also be considered. Video consultations took longer than in-person visits and required the presence of both a pediatrician and a nurse, which may not be feasible in all settings and could increase the workload. Economic, organizational, and technological implications were not evaluated in this study, and the environmental impact of digital health equipment warrants further attention.

Finally, data were collected in a single rural primary care center, which may limit the generalizability to other health care settings. Despite strict data protection measures, concerns about confidentiality and the potential for digital care to reduce the human component of clinical interaction remain relevant considerations.

### Future Directions

Future research should focus on defining evidence-based triage and feasibility criteria to select patients who can be safely and effectively managed through video interconsultation. Larger multicenter studies are needed to confirm diagnostic performance and satisfaction outcomes across broader pediatric populations and health care contexts. Evaluating economic, organizational, and environmental sustainability will be essential to inform real-world implementation. In addition, exploring strategies to streamline the workflow, optimize technical reliability, and maintain the human aspects of care will help ensure successful and responsible integration into routine pediatric practice.

### Conclusions

The proposed model of synchronous video consultation between health care professionals, including physical examination, has proven to be a feasible option. It shows good diagnostic agreement with in-person visits and has been positively evaluated by both users and health care professionals.

This approach may serve as a valuable tool for managing acute pediatric conditions in rural primary care settings in Catalonia, provided it is implemented appropriately and maintains patient safety and quality of care. Although it cannot replace in-person visits, it can complement them within the ongoing reorganization of pediatric primary care, contributing to improved accessibility, territorial equity, and system efficiency.

The implementation of this model involves several challenges, including longer consultation times, training requirements, the development of standardized protocols, economic and environmental costs, and the management of data confidentiality.

## Supplementary material

10.2196/82133 Multimedia Appendix 1 Specific reasons for consultation for each organ system.

10.2196/82133 Multimedia Appendix 2 Frequencies and diagnostic performance metrics of video interconsultations by condition, with in-person diagnoses used as the gold standard.

## Abbreviations

CONSORT: Consolidated Standards of Reporting Trials
STROBE: Strengthening the Reporting of Observational Studies in Epidemiology

## References

[1] (2022). Proposta sobre el model d’atenció pediàtrica a l’atenció primària a Catalunya. Societat Catalana de Pediatria Secció d’Atenció Primària.

[2] Rodrigo MA, Sanz AC, Pina CS (2021). The role of pediatricians in providing greater-quality care for children: an ongoing debate. J Pediatr.

[3] Aparicio Rodrigo M, Ruiz Canela J, Buñuel Álvarez JC (2020). Paediatricians provide higher quality care to children and adolescents in primary care: a systematic review. Acta Paediatr.

[4] Dall’Oglio I, Rosati GV, Biagioli V (2021). Pediatric nurses in pediatricians’ offices: a survey for primary care pediatricians. BMC Fam Pract.

[5] Societat Catalana de Pediatria. Secció d’Atenció Primària (2020). Pediatria d’Atenció primària en fallida. PediatresAP.cat.

[6] van Esso D, del Torso S, Hadjipanayis A, Primary-Secondary Working Group (PSWG) of European Academy of Paediatrics (EAP) (2010). Paediatric primary care in Europe: variation between countries. Arch Dis Child.

[7] Neumeier S (2024). Pediatric care proximity in Germany: a comparative study of regional accessibility. KN J Cartogr Geogr Inf.

[8] Bettenhausen JL, Winterer CM, Colvin JD (2021). Health and poverty of rural children: an under-researched and under-resourced vulnerable population. Acad Pediatr.

[9] Saigí-Rubió F, Vidal-Alaball J, Torrent-Sellens J (2021). Determinants of Catalan public primary care professionals’ intention to use digital clinical consultations (eConsulta) in the post-COVID-19 context: optical illusion or permanent transformation?. J Med Internet Res.

[10] Jiménez-Rodríguez D, Santillán García A, Montoro Robles J, Rodríguez Salvador MDM, Muñoz Ronda FJ, Arrogante O (2020). Increase in video consultations during the COVID-19 pandemic: healthcare professionals’ perceptions about their implementation and adequate management. Int J Environ Res Public Health.

[11] (2025). Firefly telemedicine. Firefly Global.

[12] (2025). TytoCare for professionals: advanced telemedicine solutions. TytoCare.

[13] (2025). Higo: a small medical device and telemedical solution. Higo sense.

[14] Vidal-Alaball J, Descals Singla E (2021). Abordaje de la telemedicina entre proveedores: ejemplos de uso. Atenc Prim Práct.

[15] Castillo-Rodenas M, Vidal-Alaball J, Solanas-Bacardit N (2024). Feasibility of a pediatric acute video consultation process among health care professionals in primary care in a rural setting: protocol for a prospective validation study. JMIR Res Protoc.

[16] von Elm E, Altman DG, Egger M (2008). The Strengthening the Reporting of Observational Studies in Epidemiology (STROBE) statement: guidelines for reporting observational studies. J Clin Epidemiol.

[17] Bossuyt PM, Reitsma JB, Bruns DE (2015). STARD 2015: an updated list of essential items for reporting diagnostic accuracy studies. BMJ.

[18] Eysenbach G, CONSORT-EHEALTH Group (2011). CONSORT-EHEALTH: improving and standardizing evaluation reports of web-based and mobile health interventions. J Med Internet Res.

[19] Marrugat J (2022). Calculadora de grandària mostral GRANMO. DATARUS – Applications for Biomedical Research.

[20] Vidal-Alaball J, Flores Mateo G, Garcia Domingo JL (2020). Validation of a short questionnaire to assess healthcare professionals’ perceptions of asynchronous telemedicine services: the Catalan version of the health optimum telemedicine acceptance qestionnaire. Int J Environ Res Public Health.

[21] Telehealth satisfaction questionnaire (TSQ). Agency for Healthcare Research and Quality.

[22] Casey SD, Huang J, Parry DD, Lieu TA, Reed ME (2024). Health care utilization with telemedicine and in-person visits in pediatric primary care. JAMA Health Forum.

[23] Southgate G, Yassaee AA, Harmer MJ, Livesey H, Pryde K, Roland D (2022). Use of telemedicine in pediatric services for 4 representative clinical conditions: scoping review. J Med Internet Res.

[24] Pathak PR, Stockwell MS, Lane MM (2024). Access to primary care telemedicine and visit characterization in a pediatric, low-income, primarily Latino population: retrospective study. JMIR Pediatr Parent.

[25] Wagner R, Lima TC, Silva M da (2023). Assessment of pediatric telemedicine using remote physical examinations with a mobile medical device: a nonrandomized controlled trial. JAMA Netw Open.

[26] Gao Y, Magin P, Tapley A (2025). Prevalence of antibiotic prescribing for aute respiratory tract infection in telehealth versus face-to-face consultations: cross-sectional analysis of general practice registrars’ clinical practice. J Med Internet Res.

[27] Bittmann S, Moschüring-Alieva E, Bittmann L, Luchter E, Villalon G (2021). Preliminary results of a telemedicine questionnaire in pediatrics as an innovative new tool to diagnose and treat children virtually in an ambulatory setting: analysis of 400 pediatric consultations. J Regen Biol Med.

[28] García Ron A, Arias Vivas E, Martínez del Río C (2022). Usefulness of telemedicine in urgent pediatric care during the COVID-19 pandemic [article in Spanish]. Rev Pediatr Aten Primaria.

[29] Mukusheva Z, Assylbekova M, Poddighe D (2020). Management of pediatric rheumatic patients in Kazakhstan during the coronavirus disease 2019 (COVID-19) pandemic. Rheumatol Int.

[30] Moltó-Puigmartí C, Segur-Ferrer J, Berdún Peñato J (2022). Evaluación de la seguridad, eficacia/efectividad y eficiencia de la teleconsulta en atención primaria, y de los aspectos organizativos, éticos, sociales y legales ligados a su uso. Scientia.

[31] Kodjebacheva GD, Culinski T, Kawser B, Coffer K (2023). Satisfaction with telehealth services compared with nontelehealth services among pediatric patients and their caregivers: systematic review of the literature. JMIR Pediatr Parent.

[32] (2015). Carta de drets i deures de la ciutadania en relació amb la salut i l’atenció sanitària. Departament de salut.

[33] Martín-Masot R, Diaz-Martin JJ, Santamaría-Orleans A, Navas-López VM (2023). Impact of the COVID-19 pandemic on the digitization of routine pediatric practice in Spain: a nationwide survey study. Front Pediatr.

[34] Navarro-Martínez O, Martinez-Millana A, Traver V (2024). Use of tele-nursing in primary care: a qualitative study on its negative and positive aspects. Aten Primaria.

[35] Vidal-Alaball J, López Seguí F, Garcia Domingo JL (2020). Primary care professionals’ acceptance of medical record-based, store and forward provider-to-provider telemedicine in Catalonia: results of a web-based survey. Int J Environ Res Public Health.

[36] Inoue Y, Kishi T, Sato T (2025). Telemedicine for paediatric rheumatic diseases in Japan: a national survey of physicians’ perspectives. Mod Rheumatol.

[37] Finkelstein JB, Tremblay ES, Van Cain M (2021). Pediatric clinicians’ use of telemedicine: qualitative interview study. JMIR Hum Factors.

[38] Zafra RP, Parramon NA, Albiol-Perarnau M, Torres OY (2024). Análisis de retos y dilemas que deberá afrontar la bioética del siglo XXI, en la era de la salud digital. Atención Primaria.

[39] Telemedicina: com i quan utilitzar-la en la pràctica assistencial quaderns de la bona praxi. Issuu.

