# Supplementary material for: Feasibility, Diagnostic Accuracy, and Satisfaction of an Acute Pediatric Video Interconsultation Model in Rural Primary Care in Catalonia: Prospective Observational Study
Source: JMIR Pediatr Parent. 2026 Jan 26;9:e82133. doi: 10.2196/82133 (PMC12836164; doi:10.2196/82133)
Supplement: Multimedia Appendix 1 [file pediatrics-v9-e82133-s001.docx]

This is a Multimedia Appendix to a full manuscript published in the J Med Internet Res. For full copyright and citation information see <http://dx.doi.org/10.2196/jmir.82133>

**Multimedia Appendix.** Specific reasons for consultation for each organ system (N = 200).

| **Characteristic** | | **Participants** |
| --- | --- | --- |
| **Specific consultation reasons, n (%)** | |  |
|  | Cough | 53 (26.5%) |
|  | Otalgia | 24 (12%) |
|  | Skin lesions | 23 (11.5%) |
|  | Fever | 21 (10.5%) |
|  | Odynophagia | 19 (9.5%) |
|  | Abdominal pain | 8 (4%) |
|  | Pain in the extremities | 8 (4%) |
|  | Rhinorrhea | 8 (4%) |
|  | Eye problems | 7 (3.5%) |
|  | Headache | 4 (2%) |
|  | Genital problem | 4 (2%) |
|  | Vomiting | 2 (1%) |
|  | Dysphonia | 2 (1%) |
|  | Oral pain | 2 (1%) |
|  | Back pain | 2 (1%) |
|  | Contusion | 2 (1%) |
|  | Respiratory distress | 2 (1%) |
|  | Chest pain | 1 (0.5%) |
|  | Neck pain | 1 (0.5%) |
|  | Hearing loss | 1 (0.5%) |
|  | Dizziness | 1 (0.5%) |
|  | Mouth ulcer | 1 (0.5%) |
|  | Diarrhea | 1 (0.5%) |
|  | Other | 3 (1.5%) |
