# Supplementary material for: Feasibility, Diagnostic Accuracy, and Satisfaction of an Acute Pediatric Video Interconsultation Model in Rural Primary Care in Catalonia: Prospective Observational Study
Source: JMIR Pediatr Parent. 2026 Jan 26;9:e82133. doi: 10.2196/82133 (PMC12836164; doi:10.2196/82133)
Supplement: Multimedia Appendix 2 [file pediatrics-v9-e82133-s002.docx]

This is a Multimedia Appendix to a full manuscript published in the J Med Internet Res. For full copyright and citation information see <http://dx.doi.org/10.2196/jmir.82133>

## **Multimedia Appendix. Table full results diagnostic performance**

Frequencies and diagnostic performance metrics (accuracy, sensitivity, and specificity) of video interconsultations by condition, with in-person diagnoses used as the gold standard.

| **Specific**  **diagnosis** | **Video consultation**  **N (%)** | **In-person visit**  **N (%)** | **Accuracy**  **[95% CI]** | **Sensitivity**  **[95% CI]** | **Specificity**  **[95% CI]** |
| --- | --- | --- | --- | --- | --- |
| Abdominal pain | 4 (2%) | 1 (0.5%) | 0.98 [0.96–1.00] | 1.00 [0.02–1.00] | 0.98 [0.96–1.00] |
| Acute gastroenteritis | 4 (2%) | 6 (3%) | 0.99 [0.96–1.00] | 0.67 [0.22–0.96] | 1.00 [0.98–1.00] |
| Acute otitis media | 17 (8.5%) | 16 (8%) | 0.97 [0.95–0.99] | 0.93 [0.66–1.00] | 0.98 [0.95–0.99] |
| Acute pharyngitis | 10 (5%) | 10 (5%) | 1.00 [0.98–1.00] | 1.00 [0.69–1.00] | 1.00 [0.98–1.00] |
| Adenopathy | 1 (0.5%) | 1 (0.5%) | 1.00 [0.98–1.00] | 1.00 [0.02–1.00] | 1.00 [0.98–1.00] |
| Ankle sprain | - | 1 (0.5%) | - | - | - |
| Atopic dermatitis | 3 (1.5%) | 4 (2%) | 0.99 [0.97–1.00] | 0.75 [0.19–1.00] | 1.00 [0.98–1.00] |
| Balanitis | 3 (1.5%) | 3 (1.5%) | 1.00 [0.98–1.00] | 1.00 [0.29–1.00] | 1.00 [0.98–1.00] |
| Bite | 1 (0.5%) | 1 (0.5%) | 1.00 [0.98–1.00] | 1.00 [0.02–1.00] | 1.00 [0.98–1.00] |
| Bone protrusion | 1 (0.5%) | - | - | - | - |
| Bronchiolitis | 3 (1.5%) | 4 (2%) | 0.99 [0.97–1.00] | 0.75 [0.19–0.99] | 1.00 [0.98–1.00] |
| Bronchospasm | 16 (8%) | 21 (10.5%) | 0.95 [0.92–0.98] | 0.67 [0.43–0.85] | 0.99 [0.97–1.00] |
| Capsulitis | 1 (0.5%) | 2 (1%) | 0.99 [0.97–1.00] | 0.50 [0.01–0.99] | 1.00 [0.98–1.00] |
| Cerumen impaction | 1 (0.5%) | 1 (0.5%) | 1.00 [0.98–1.00] | 1.00 [0.02–1.00] | 1.00 [0.98–1.00] |
| Conjunctivitis | 7 (3.5%) | 7 (3.5%) | 1.00 [0.98–1.00] | 1.00 [0.59–1.00] | 1.00 [0.98–1.00] |
| Constipation | - | 1 (0.5%) |  |  |  |
| Contusion | 5 (2.5%) | 4 (2%) | 0.99 [0.96–1.00] | 1.00 [0.20–1.00] | 0.99 [0.96–1.00] |
| Cough | 2 (1%) | 2 (1%) | 1.00 [0.98–1.00] | 1.00 [0.16–1.00] | 1.00 [0.98–1.00] |
| Dental abscess | - | 1 (0.5%) |  |  |  |
| Enterovirus infection | - | 1 (0.5%) |  |  |  |
| Erythema infectiosum | 1 (0.5%) | 1 (0.50%) | 1.00 [0.98–1.00] | 1.00 [0.02–1.00] | 1.00 [0.98–1.00] |
| Fever | 1 (0.5%) | - | - | - | - |
| Hand, foot, and mouth | 1 (0.5%) | 1 (0.50%) | 1.00 [0.98–1.00] | 1.00 [0.02–1.00] | 1.00 [0.98–1.00] |
| Headache | 1 (0.5%) | - | - | - | - |
| Heel pain | 1 (0.5%) | - | - | - | - |
| Hematoma | 1 (0.5%) | 1 (0.50%) | 1.00 [0.98–1.00] | 1.00 [0.02–1.00] | 1.00 [0.98–1.00] |
| Herpangina | - | 3 (1.50%) |  |  |  |
| Herpes simplex | 1 (0.5%) | 1 (0.50%) | 1.00 [0.98–1.00] | 1.00 [0.02–1.00] | 1.00 [0.98–1.00] |
| Herpes zoster | 1 (0.5%) | 1 (0.50%) | 1.00 [0.98–1.00] | 1.00 [0.02–1.00] | 1.00 [0.98–1.00] |
| Impetigo | 5 (2.5%) | 6 (3.00%) | 1.00 [0.98–1.00] | 1.00 [0.48–1.00] | 1.00 [0.98–1.00] |
| Influenza | 1 (0.5%) | 1 (0.50%) | 1.00 [0.98–1.00] | 1.00 [0.02–1.00] | 1.00 [0.98–1.00] |
| Laryngitis | 5 (2.5%) | 5 (2.50%) | 1.00 [0.98–1.00] | 1.00 [0.48–1.00] | 1.00 [0.98–1.00] |
| Mouth ulcer | 3 (1.5%) | 3 (1.50%) | 1.00 [0.98–1.00] | 1.00 [0.29–1.00] | 1.00 [0.98–1.00] |
| Muscle tightness | - | 1 (0.50%) |  |  |  |
| Myalgia | 5 (2.5%) | 3 (1.50%) | 0.99 [0.96–1.00] | 1.00 [0.29–1.00] | 0.99 [0.96–1.00] |
| Orthostatic hypotension | 1 (0.5%) | 1 (0.50%) | 1.00 [0.98–1.00] | 1.00 [0.02–1.00] | 1.00 [0.98–1.00] |
| Otalgia | 5 (2.5%) | 3 (1.50%) | 0.98 [0.95–0.99] | 0.67 [0.09–0.99] | 0.98 [0.96–1.00] |
| Otitis externa | 2 (1%) | 4 (2.00%) | 0.99 [0.96–1.00] | 0.50 [0.07– 0.93] | 1.00 [0.98–1.00] |
| Ovulation | - | 1 (0.50%) |  |  |  |
| Pectus carinatum | - | 1 (0.50%) |  |  |  |
| Perioral dermatitis | 2 (1%) | 2 (1.00%) | 1.00 [0.98–1.00] | 1.00 [0.16–1.00] | 1.00 [0.98–1.00] |
| Pityriasis rosea | 1 (0.5%) | 1 (0.50%) | 1.00 [0.98–1.00] | 1.00 [0.02–1.00] | 1.00 [0.98–1.00] |
| Pneumonia | 3 (1.5%) | 6 (3.00%) | 0.99 [0.96–1.00] | 1.00 [0.02–1.00] | 0.99 [0.96–1.00] |
| Respiratory superinfection | 2 (1%) | 3 (1.5%) | 0.98 [0.96–1.00] | 0.33 [0.01–0.99] | 0.99 [0.96–1.00] |
| Scabies | 1 (0.5%) | 1 (0.5%) | 1.00 [0.98–1.00] | 1.00 [0.02–1.00] | 1.00 [0.98–1.00] |
| Scarlet fever | 1 (0.5%) | 2 (1%) | 0.99 [0.97–1.00] | 0.50 [0.01–0.99] | 1.00 [0.98–1.00] |
| Serous otitis media | 2 (1%) | 3 (1.5%) | 0.99 [0.97–1.00] | 0.67 [0.09–0.99] | 1.00 [0.98–1.00] |
| Sever's disease | - | 1 (0.5%) |  |  |  |
| Skin lesions | 1 (0.5%) | - | - | - | - |
| Streptococcal perianal | 1 (0.5%) | 1 (0.5%) | 1.00 [0.98–1.00] | 1.00 [0.02–1.00] | 1.00 [0.98–1.00] |
| Streptococcal pharyngitis | 5 (2.5%) | 5 (2.5%) | 1.00 [0.98–1.00] | 1.00 [0.29–1.00] | 1.00 [0.98–1.00] |
| Tonsillar hypertrophy | 1 (0.5%) | 1 (0.5%) | - | - | - |
| Tooth contusion | 1 (0.5%) | 1 (0.5%) | 1.00 [0.98–1.00] | 1.00 [0.02–1.00] | 1.00 [0.98–1.00] |
| Toothache | 1 (0.5%) | - | - | - | - |
| Upper respiratory infection | 55 (27.5%) | 42 (21%) | 0.92 [0.87–0.95] | 0.95 [0.84–0.99] | 0.91 [0.85–0.95] |
| Urticaria | 2 (1%) | 2 (1%) | 1.00 [0.98–1.00] | 1.00 [0.16–1.00] | 1.00 [0.98–1.00] |
| Viral infection | 4 (2%) | 4 (2%) | 0.99 [0.96–1.00] | 0.75 [0.19–0.99] | 0.99 [0.97–1.00] |
| Vomiting | 2 (1%) | 1 (0.5%) | 0.99 [0.97–1.00] | 1.00 [0.02–1.00] | 0.99 [0.98–1.00] |
| Vulvitis | 1 (0.5%) | 1 (0.5%) | 1.00 [0.98–1.00] | 1.00 [0.02–1.00] | 1.00 [0.98–1.00] |
| **Overall mean** | | | **0.99 [0.98–1.00]** | **0.90 [0.84–0.95]** | **0.99 [0.98–1.00]** |

Validation indicators could not be calculated in empty cells due to a lack of available cases. In one instance, two diagnoses were made during the same in-person visit, whereas only one was recorded during the corresponding video consultation. Therefore, the total number of cases in the video consultation column is 200, compared with 201 in the in-person visit column.
